# Comparison of machine learning and deep learning-based methods for locomotion mode recognition using a single inertial measurement unit

**DOI:** 10.3389/fnbot.2022.923164

**Published:** 2022-11-29

**Authors:** Huong Thi Thu Vu, Hoang-Long Cao, Dianbiao Dong, Tom Verstraten, Joost Geeroms, Bram Vanderborght

**Affiliations:** ^1^Brubotics, Vrije Universiteit Brussel and imec, Brussels, Belgium; ^2^Faculty of Electronics Engineering Technology, Hanoi University of Industry, Hanoi, Vietnam; ^3^Brubotics, Vrije Universiteit Brussel and Flanders Make, Brussels, Belgium; ^4^College of Engineering Technology, Can Tho University, Can Tho, Vietnam; ^5^School of Mechanical Engineering, Northwestern Polytechnical University, Xi'an, China

**Keywords:** locomotion mode recognition, lower limb prosthesis, transportation mode classification, deep learning-artificial neural network (DL-ANN), machine learning, wearable device and IMU sensor, assistive devices

## Abstract

Locomotion mode recognition provides the prosthesis control with the information on when to switch between different walking modes, whereas the gait phase detection indicates where we are in the gait cycle. But powered prostheses often implement a different control strategy for each locomotion mode to improve the functionality of the prosthesis. Existing studies employed several classical machine learning methods for locomotion mode recognition. However, these methods were less effective for data with complex decision boundaries and resulted in misclassifications of motion recognition. Deep learning-based methods potentially resolve these limitations as it is a special type of machine learning method with more sophistication. Therefore, this study evaluated three deep learning-based models for locomotion mode recognition, namely recurrent neural network (RNN), long short-term memory (LSTM) neural network, and convolutional neural network (CNN), and compared the recognition performance of deep learning models to the machine learning model with random forest classifier (RFC). The models are trained from data of one inertial measurement unit (IMU) placed on the lower shanks of four able-bodied subjects to perform four walking modes, including level ground walking (LW), standing (ST), and stair ascent/stair descent (SA/SD). The results indicated that CNN and LSTM models outperformed other models, and these models were promising for applying locomotion mode recognition in real-time for robotic prostheses.

## 1. Introduction

Able-bodied walkers can simply adjust their movements in response to the transitions in different environments they encounter during daily activities. It is a huge challenge for amputees to cope with these topographic changes. Because they lost all sensory feedback from their foot-ankle that are necessary to deal with the transitions between different walking modes, such as flat walking, stop walking, going up/down stairs, and walking on slopes (Hansen et al., 2004; Prentice et al., 2004). Additionally, the walking of amputees costs remarkably more metabolic energy than that of healthy persons, resulting in slower walking speeds, asymmetric gait patterns, and decreased stride length compared to the non-disabled at the same velocities (Herr and Grabowski, 2012; Ledoux et al., 2015).

In the field of prosthesis design, researchers have been developing active foot prosthetics for reproducing the dynamic functions of the limbs for the prosthetic wearers. Different methods to detect gait phases and events were embedded in the prosthetic control strategies to increase the accuracy of walking. Xu et al. (2020) developed an algorithm online for the estimation of gait phase based on capacitive sensing signals, which could be applied to their robotic prosthesis to detect two phases. This method can improve the detection accuracy and the smoothness of the transition timing between two different gait phases of the steps. Vu et al. (2018) introduced an algorithm for gait percent prediction where a full gait cycle discretized 100% within one cycle is predicted. Flynn et al. (2018) presented a prosthesis with high-level control which was directed by a wrist-worn touchscreen system. This system allowed the pilot to select the high-level behavior of the machine that was in operation. Each of these state machines consisted of trajectory generators for the knee actuator, ankle actuator, and weight acceptance systems.

The prosthetic control programs were embedded with phase and event detection and locomotion recognition algorithms for walking in different terrains. The prosthesis can automatically switch safely and smoothly between different control modes. Therefore, many prosthetic research teams have focused on developing the adaptation of the prostheses' behaviors as locomotion assistance scenarios when amputees transition from different locomotion modes.

The performance outcomes of these locomotion recognition systems vary depending on the types of sensors used, and the algorithms applied. Al-dabbagh and Ronsse (2020) categorized typical sensors that were employed for locomotion recognition systems, both exteroceptive sensors and proprioceptive sensors. Exteroceptive sensors, such as vision or ranging sensors, measure the external environment to obtain the distances or the depth of the object. Proprioceptive sensors capture the body's movements to provide information on the internal state of the body. For instance, IMUs, electromyography (EMG), force sensors, and pressure sensors-based systems are currently the most popular choices as EMG is a lightweight and inexpensive wearable sensor that specifically measures electrical muscle activities produced by muscle movements when performing tasks. EMG-based recognition studies produced high accuracy (Huang et al., 2008; Pati et al., 2010; Kim et al., 2014). However, the limitation of using this sensor requires sensors to be integrated directly into the skin (Chen et al., 2013). Therefore, EMG signals could be affected by environmental noise, shifts in electrode positions, or even the loss of electrode sensor contact caused by the moisture between sensors and the skin such as sweating and humidity (Rafiee et al., 2011; Phinyomark et al., 2013; Taborri et al., 2016). For instance, Huang et al. (2008) presented a system that using 16 EMGs that could distinguish between seven gait modes. However, their approach suffered from EMG signal variations caused by physical changes, which resulted in decreasing recognition performance over time, especially in locomotion mode recognition for walking outside the laboratory conditions. Using pressure-sensitive insoles is another approach for locomotion mode recognition with a simple method. This sensor measures the reaction force between the human body and the ground (Chen et al., 2014, 2015; Parri et al., 2017; Godiyal et al., 2018). The limitation of these insole sensors is that their signals are affected by the sensor positions. Additionally, this sensor is sensible to mechanical failure given the high and repeated impact forces occurring between the foot and the ground (Novak et al., 2014; Tiwari and Joshi, 2020). IMU sensors are the most widely used for all rehabilitation applications it provides accelerations and angular velocities of the body part where they are placed to capture the movements of the human walk (Ahmad et al., 2013; Young et al., 2014; Zhang et al., 2014). Besides, IMU sensors are compact, low-energy, enduring, and stable to measure the gait data in various environments with high precision characteristics of gait signals (Ahmad et al., 2013; Taborri et al., 2016; Vu et al., 2020). Furthermore, the information of IMU signals is applicable to machine learning-based recognition approaches, allowing the algorithm to gain high performance of recognition (Kim et al., 2019; Hu et al., 2021). Due to the above reasons, the use of IMU sensors for the recognition system proposes three criteria of prosthetic design: electric battery efficiency, sensor durability, and aesthetics (Ahmad et al., 2013). Hence, IMU sensor is commonly used for the rehabilitation fields, especially in practical applications, and potentially applied for commercial prostheses (Ledoux, 2018).

Labarrière et al. (2020) reported that various locomotion recognition systems applied either classical machine learning or pattern recognition techniques. Machine learning-based method involves numerous classifiers such as decision tree (Liu et al., 2015; Han et al., 2021), random forest classifier (RFC) (Billah et al., 2019), linear discriminant analysis (LDA) (Hargrove et al., 2013; Tkach and Hargrove, 2013; Chen et al., 2015; Liu et al., 2016), support vector machines (SVM) (Huang et al., 2011; Long et al., 2016; Zhou et al., 2016; Ai et al., 2017; Tiwari and Joshi, 2020), quadratic discriminant analysis (QDA) (Bhakta et al., 2020), XGBoost (Bhakta et al., 2020), and artificial neural networks (ANN) (Woodward et al., 2016; Hu et al., 2018a; Ma et al., 2020), as well as a type of ANN is back propagation neural network (BPNN) (Gong et al., 2020). Although these methods have demonstrated the advantages, such as ease of use and fast training, by exploring data structures and mapping functions, they are limited in capturing complex data dependencies resulting in misclassifications. In addition, classical machine learning models perform well only with certain movement events such as heel strike or toe off. Therefore, typical machine learning models for locomotion mode detection require the use of mechanical sensors as force sensors, loadcells, or pressure sensors to achieve better accuracy. We summarize existing studies that employ machine learning techniques in **Table 5**. Recently, deep learning approaches overcome the limitations of basic machine learning methods due to their effectiveness. Among hundreds of deep learning methods, some deep learning models have been focused on locomotion mode recognition. For instance, recurrent neural networks (RNN) and the long short-term memory (LSTM) are among the most promising models since they can obtain excellent performance in learning time-series signals (Wang et al., 2018; Lu et al., 2020a). The convolutional neural network (CNN) has been employed regularly for terrain mode classification and human activity recognition because it could learn features automatically from simple to complex data by complicated layer-by-layer structures, also from raw sensor signal inputs (Su et al., 2019; Lu et al., 2020b; Tiwari and Joshi, 2020; Narayan et al., 2021). **Tables 4, 5** show the categorization of existing studies that used basic machine learning and deep learning models based on an outline of the material and methods and the accuracies of these studies. More deep learning studies have been applied in recent years due to their higher performance. While these existing studies installed many sensors in their systems to take advantage of only IMU sensor signals, we aim to reduce the number of sensors by using only 6-axis IMU to save the space of sensor positions and the cost of the prosthetic design. The contributions of this research are as follows:

1. Machine learning and deep learning-based methods are implemented to classify and detect four locomotion modes, i.e., level ground walking (LW), standing (ST), and stair ascent/stair descent (SA/SD). We implement both traditional machine learning algorithms and deep learning models.
2. This paper compares the performance of different deep learning model approaches for the further development of rehabilitation applications and prosthetic designs.

This paper is organized into four sections. Section 1 introduces the work and related studies. Section 2 proposes the methodology and different models for locomotion mode recognition. Section 3 presents the details of materials and experimental setups. Section 4 shows the results and discussion. Section 5 provides the conclusion.

## 2. Methods

Deep learning techniques are now commonly employed in all fields and research areas (Sarker, 2021). There are various open-source Python Libraries and Frameworks for machine learning, such as TensorFlow, Keras, and Matplotlib. This allows to develop and train the models more simply and easily (Chen et al., 2020; Labarrière et al., 2020). In this study, we applied four models of machine learning RFC, and deep learning RNN, LSTM, and CNN for locomotion mode recognition. These models were deployed from the Google Colab environment based on the open-source Keras libraries, using Python language. We aimed to produce an inexpensive system installing only IMU sensor for locomotion recognition with reliable accuracy compared to other methods.

### 2.1. Data feature extraction

**Figure 1:**
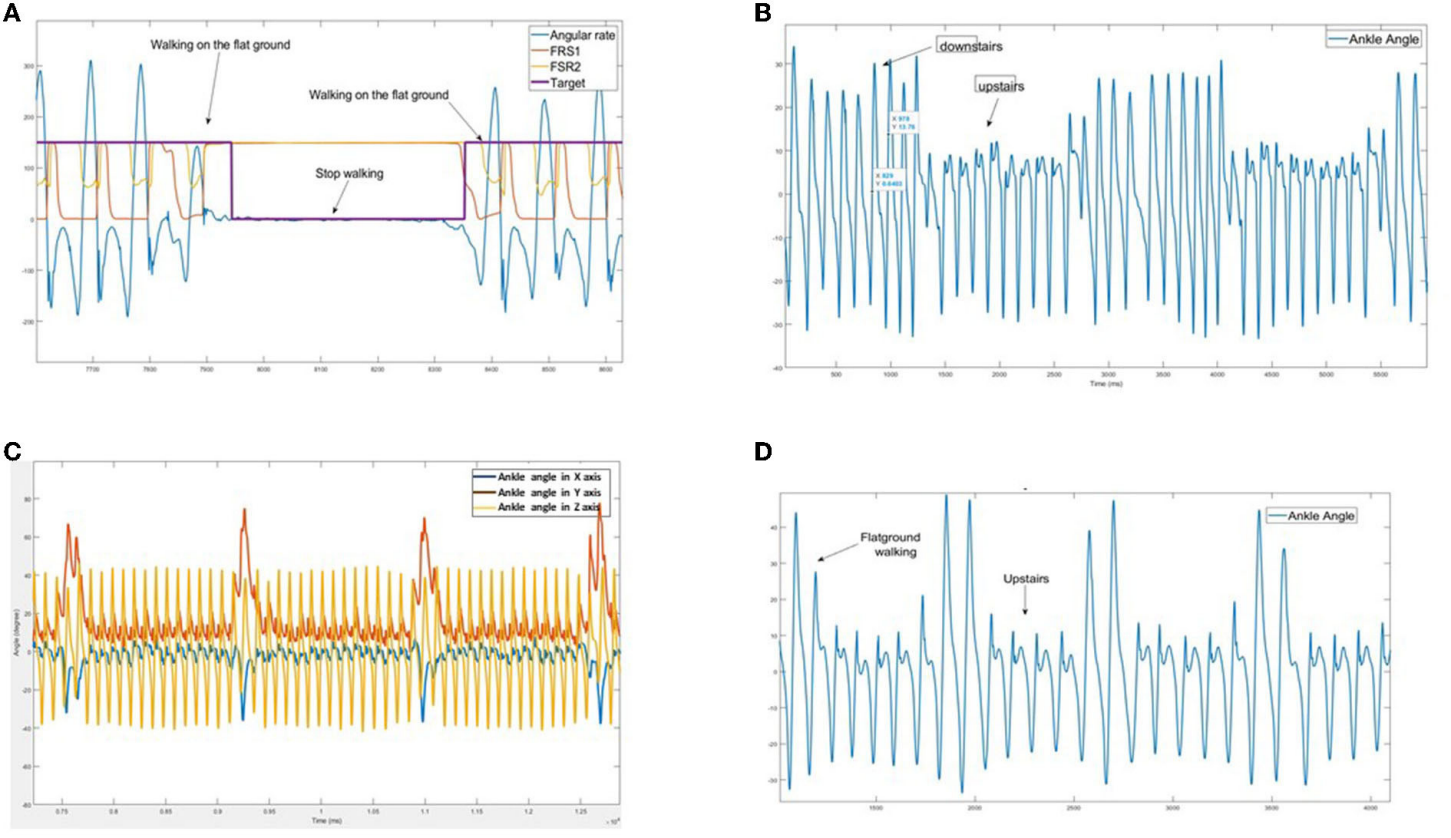
Observation signal patterns of the inertial measurement unit (IMU) on different walking surfaces: resultant angular velocity (rad/s) and Force Sensitive Resistor (FSR) sensors. The distinguished characteristics of tanks **(A)** level ground walking (LW) and standing (ST); **(B)** stair descent (SD) and stair ascent (SA); **(C)** FW and turning; **(D)** FW and SA. We use all six input signals of tri-gyroscope and tri-accelerometer for the training models; however, significant signals are depicted in this figure.

The data feature is important for the recognition algorithm. Models will extract the relevant characteristics of different locomotion modes during the training process to make predictions for unknown datasets. For instance, Figure 1 shows clearly the changes in IMU signals in different walking tasks: SD/SA and flat ground walking. This means that the IMU sensor produces distinct features of different walking modes and the transitions between them. Additionally, walking signals are in periodicity time-series, i.e., periodic and sequential. Hence, it is feasible to utilize deep learning approaches to automatically extract feature learning from input samples to predict unknown motion patterns.

### 2.2. Data labeling and processing

The dataset of locomotion mode from IMU was manually segmented into activities performed in experimental protocol and tagged with different labels for different locomotion modes as shown in **Figure 7** and Table 1. The data sequence was classified into input vectors **X** = [*x*_*t*−*k*_, ...*x*_*t*_], each vector included *k* samples in time series taken at the current time *t* and *k-1* samples in the past. This vector was also a function of the window [*x*_*t*−*k*_, ...*x*_*t*_] for the window size of *k*. The input signals were mapped output labels of sequences **Y** = [*y*_*t*−*k*_, .., *y*_*t*_] to obtain the supervised dataset. As a result, the network learned to estimate present and future outputs **Y** = [*y*_*t*_, *y*_*t*+1_, .., *y*_*t*+*n*_], where n indicates the number of consequent outputs to predict in the future.

**Table 1:** Notations used for different terrain transitions.

**Number**	**Mode transitions**	**Notation**
1	Stop walking → Level ground walking	ST → LW
2	Level ground walking → Stop walking	LW → ST
3	Level ground walking → Stair ascent	LW → SA
4	Stair ascent → Level ground walking	SA → LW
5	Level ground walking → Stair descent	LW → SD
6	Stair descent → Level ground walking	SD → LW

### 2.3. Recognition methodologies

**Figure 2:**
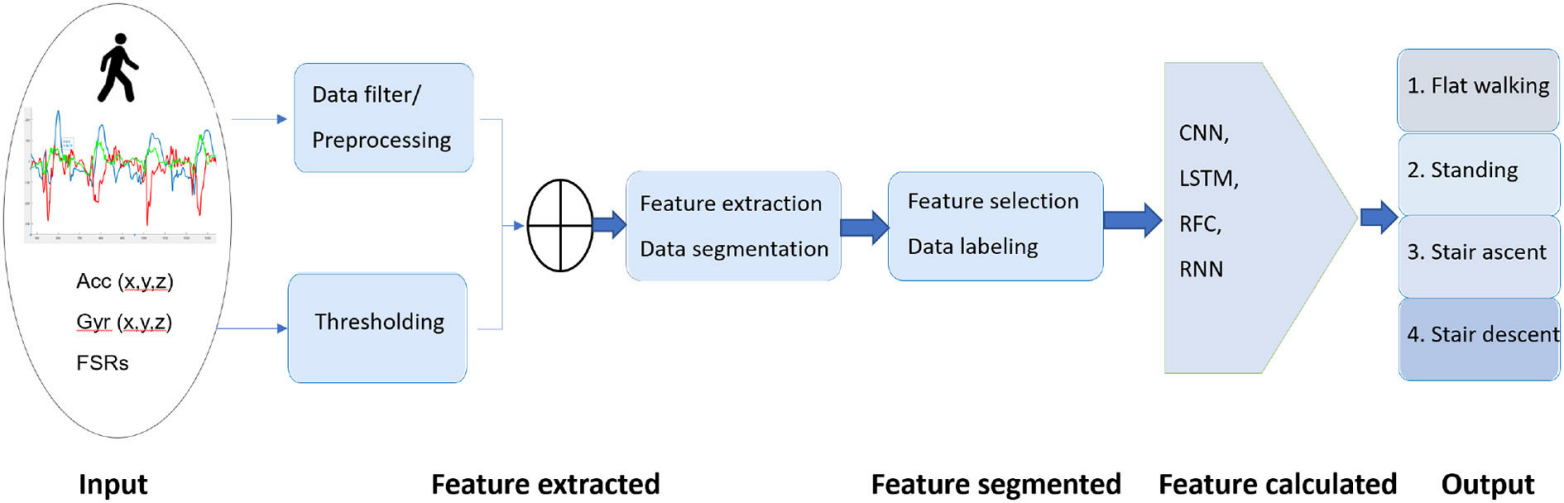
The structure of machine learning approaches. The inputs to the network are created by six signal channels of one IMU, with the length of each channel being the sliding window of sequential sample inputs. The outputs are classified into four classes of four walking modes.

The structure diagram shown in Figure 2 is used to design for both prediction and recognition purposes. In this section, representative models, i.e., RFC, RNN, LSTM, and CNN, were constructed as the target models, which were evaluated for the possibly highest performance of different deep models using the same dataset. These models are demonstrated in this section and described in detail as follows.

#### 2.3.1. Random forest classifier

**Figure 3:**
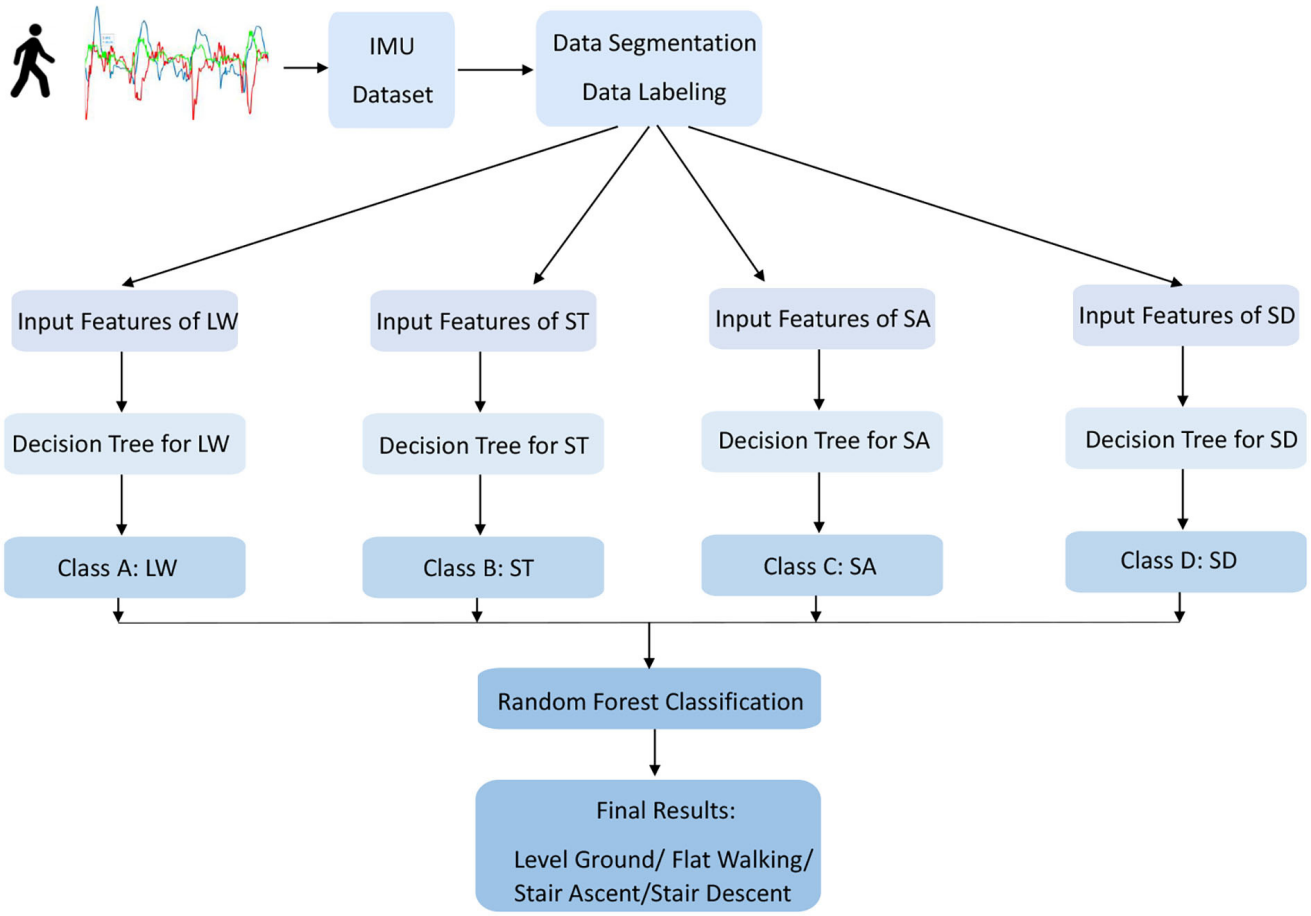
The structure of the overall random forest classifier (RFC). The inputs to the network are created by six signal channels of one IMU, and the outputs are classified into four walking modes: LW, ST, and SA/SD.

Random forest classifier is one of the classification methods which belongs to the supervised machine learning approach, as depicted in Figure 3. It is widely used for pattern recognition and segmentation problems. RFC is a strong modeling technique containing a collection of decision trees to solve a complex problem and improve the model's performance. It is more robust than a single decision tree to limit the over-fitting issue and the error due to bias. Therefore, the model enhances the accuracy of valuable results. It classifies the raw data inputs into a target category of four different locomotion modes. At the end of the learning process, the model obtains the features and classifiers distinguished between four locomotion modes in minimum time with maximum accuracy.

#### 2.3.2. Neural network model with time series

*A. RNN model training*

**Figure 4:**
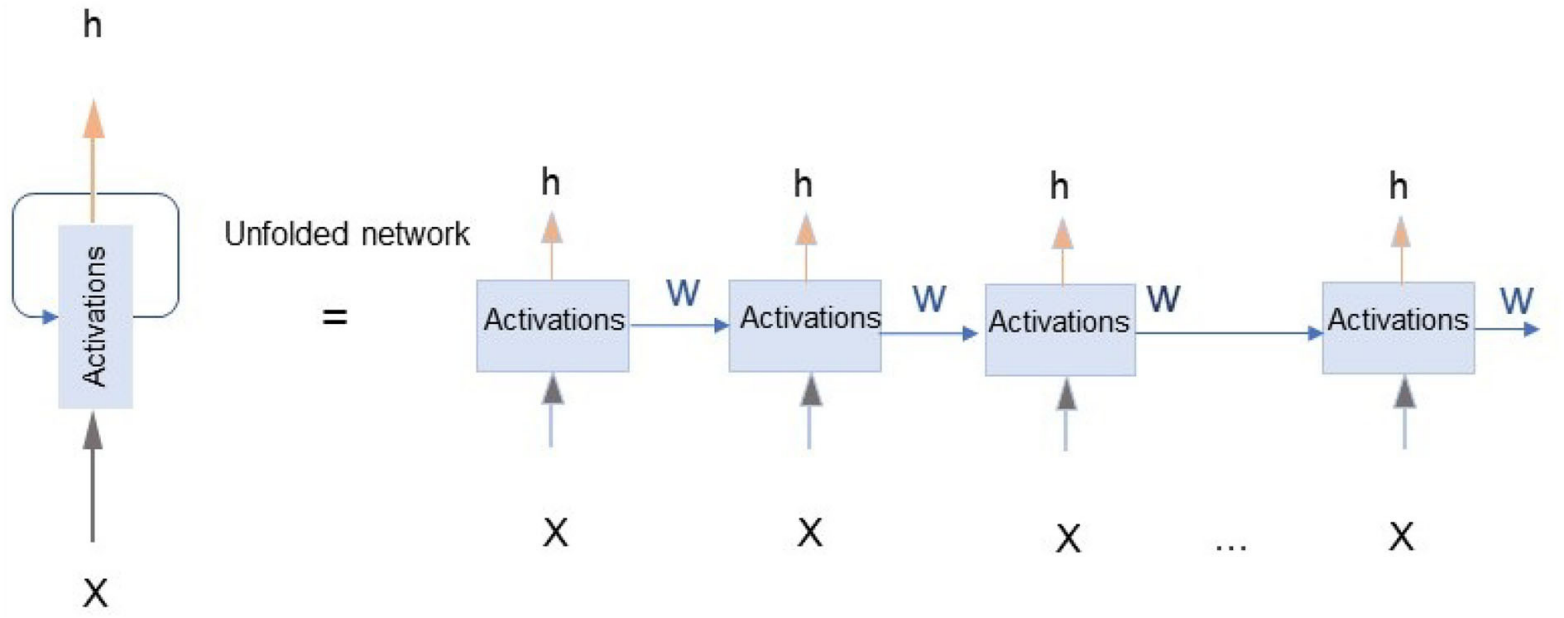
The left diagram illustrates the recurrent neural network (RNN) with an infinite loop network, with the model outputs fed back as inputs. The right figure is an unfolded representation of an RNN with X: input, h: hidden state, or output of each unit (LeCun et al., 2015).

The recurrent neural network is an extension of an ANN, which has loops in them, as shown in Figure 4. The output at time *t* of an RNN unit is computed from both the input at time *t* and output at the time *t-1*. Thus, RNN can remember the previous and the current inputs and outputs (Ranzato et al., 2015). In this experiment, we build a typical RNN having a three-layer structure: input layer, hidden layer, and output layer. The first layer is an input layer that consists of six input signals in a time series sequence. The second layer is a hidden layer that contains 6 x 30 RNN units. Additionally, there is a sigmoid activation function associated with each note. Here, the activation functions flow in only one direction, from the input layer to the output layer, where one neuron receives inputs, produces an output, and sends that output back to itself. An RNN is very much similar to a feedforward neural network. In this case, its output at time *t* is the hidden state itself.

*B. LSTM model training*

**Figure 5:**
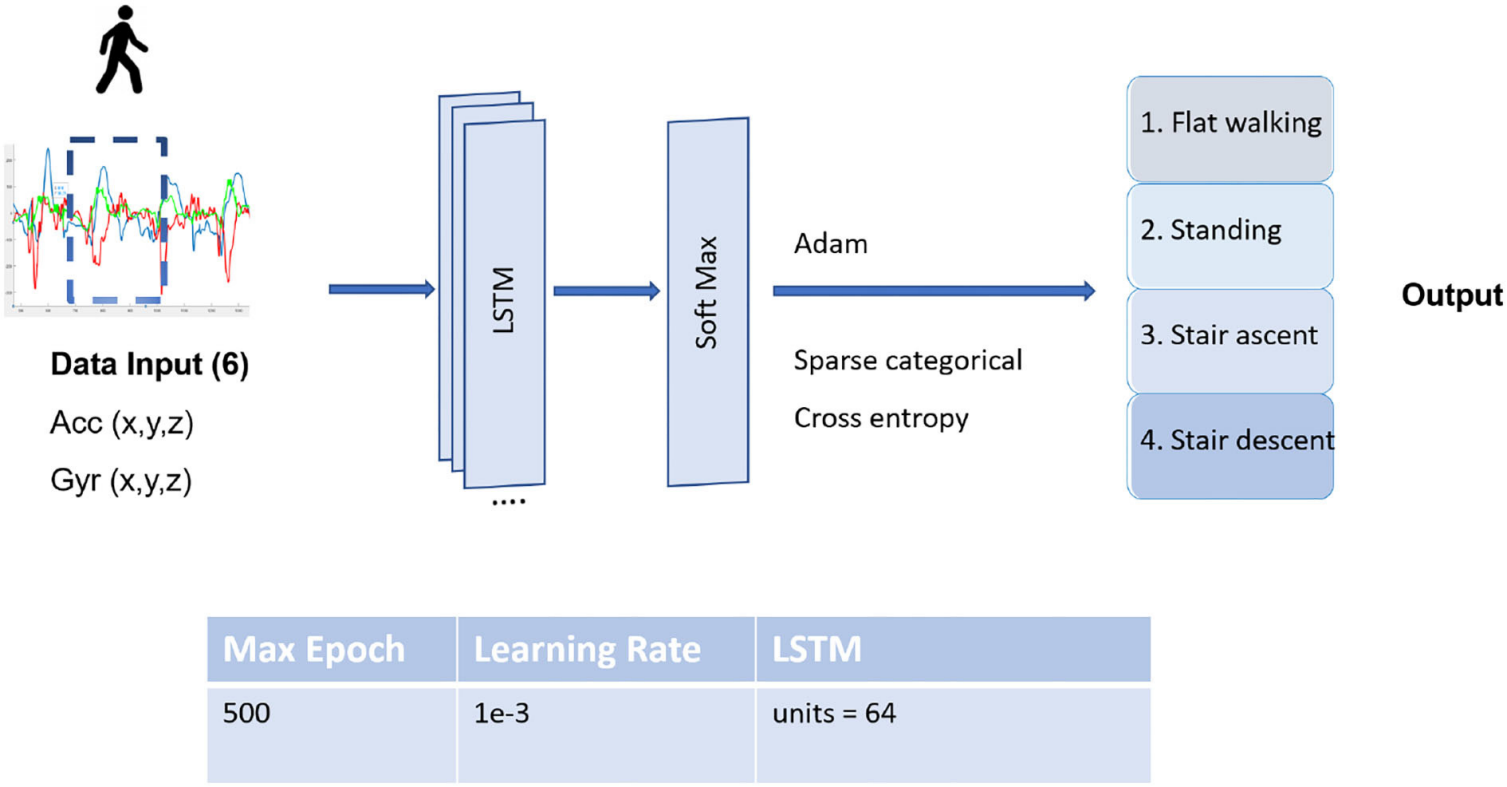
The structure of the long short-term memory (LSTM) network and its parameters. The LSTM unit is implemented as a typical LSTM cell detailed by Colah (2015).

Long short-term memory model is an advanced RNN, a sequential and time series data network for classification and regression tasks, which is currently the most suitable approach not only for locomotion mode detection but gait prediction algorithms. In this model, the dataset is trained by means of a feature selection and retrieval process to minimize categorical cross-entropy, and the LSTM prediction principle is applied. The LSTM network architecture is divided into three layers as illustrated in Figure 5. The input layer directly processes input signals in a time series sequence given by the users and forwards them to the LSTM layers that contain the LSTM nodes. The input layer calculates the weight values based on the raw signal inputs, while LSTM layers learn complex representations and more specific characteristics of the input data. The outputs of LSTM are fed into the last layer of four output notes with softmax activation functions that classify the possibility of four locomotion modes. Model weights are optimized with an ADAM optimizer algorithm and an adaptive learning rate optimization algorithm. The model finished training around 70 epochs.

The input data obtained from the accelerometer and gyroscope sensors are all three-dimensional values of the X, Y, and Z-axes. Hence, the input window sizes have the dimension of *k* time-steps multiplied by six input signals. Every time step, one input data window is slid into the LSTM model. The model was trained several times with the lengths of different size windows of 50 ms, 100 ms, or 200 ms with a 10 ms signal sampling to observe the performance results as shown in **Table 3**. The dataset from four participants is mixed all together in time series and is split into 80% for training and 20% for validation data.

*C. CNN model training*

**Figure 6:**
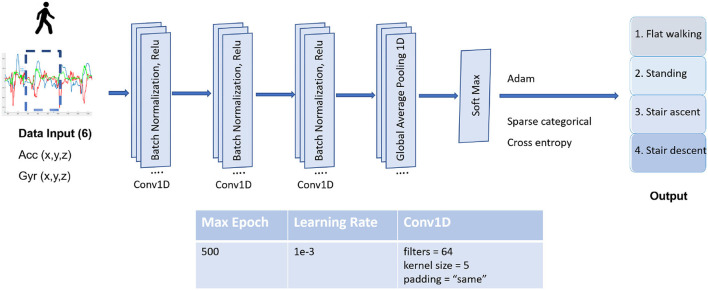
The structure of the computational neural network (CNN) and its type and parameters. 1D convolution layer (Conv1D) indicates each input channel is one dimensional with 64 filters and a kernel size of 5. The Conv1D layers use a batch normalization (BN), and rectified linear unit (ReLU) is used as an activation function.

The convolutional neural network is a feed-forward ANN that is mostly applied for image recognition and semantic segmentation. However, it was proven efficient in resolving signals with time series, given the ability to automatically extract deep features from raw data. The proposed CNN model shown in Figure 6 has three 1D convolution layers (Conv1Ds) with 64 filters and a kernel size of 5 and pooling layers. The Conv1D layers use batch normalization (BN) to make neural networks faster and more stable by adding extra layers to a deep neural network. They are followed by an activation function of a rectified linear unit (ReLU) and a max pooling layer for downsampling. At the output layer, softmax activation functions are used to produce the outputs corresponding to the output possibilities. In the end, the model is optimized with an ADAM optimizer, an adaptive learning rate optimization algorithm, and is done at 160 epochs. The input matrix has two dimensions for all models: number of features x number of samples. These models have the matrix input of 6 features with different sequence time steps of 50, 100, or 200 ms, depending on different training tests.

### 2.4. Evaluation

To verify the performance of the proposed models, we employed a traditional approach named cross-validation (Ha and Choi, 2016; Zdravevski et al., 2017; Gholamiangonabadi et al., 2020). The dataset of all participants is randomly partitioned into K groups, one of the groups is used for the test set, and the rest are used for the training set. The training set is used to train the model, and the test set is used to analyze the performance of the model on unseen data. This method indicates how well the model performs with a new unknown data set. Overrunning the model several times, we computed the accuracy and the mean loss to determine the overall effectiveness of four models by using *spare_categorical_accuracy* and *val_loss* functions of Keras library, respectively. The accuracy function is calculated as the ratio between the number of true positive predictions and true negative predictions to the total number of predictions. The loss is the sum of the squared error, which is computed as the difference between the true values and the values predicted by the model of the problem (James, 2003). The best model achieved greater accuracy, with low loss.

## 3. Experiment

This section is divided into three parts. Section 3.1 presents the hardware design used to read IMU signals. Section 3.2 describes the setup scenarios performed by the subjects. Section 3.3 explains how the signals were recorded and pre-processed.

### 3.1. Experimental electronic measurement system

We designed an electronic board for collecting the signals of human motions. One IMU sensor, two FSRs, and an Arduino board, Adafruit Feather M0 Bluefruit LE (the processor used was ATSAMD21G18 ARM Cortex M0, clocked at 48 MHz, 256K of FLASH ROM, and 32K of RAM), were embedded on this board. The IMU and two FSRs were utilized to measure the gait signals of the subjects when they walked in different scenarios. The IMU (MPU 6000-Invensense) was attached to the shank and provided a 3-axis gyroscope sensor and a 3-axis accelerometer. Moreover, the IMU data were transferred to the Arduino *via* the SPI interface for high-speed up to 1MHz. The gyroscope resolution was set at a full range scale of ±2, 000 degrees/s correspondence to a sensitivity of ±16 g LSB/degree/s, and the resolution of the accelerometer was set at a full range scale of ±16 g with a sensitivity of 2,048 LSB/g (*g* = 9.8 m/s^2^). Furthermore, the FSRs were placed in the shoe sole under the toe and the heel of the subject to detect the contact of the foot with the ground in order to label the steps of the dataset. The FSRs were used for the gait phase detection, and they were unnecessary for locomotion mode detection. All signals were recorded in synchronous intervals of 10 ms, and then transferred directly to the computer. The Arduino board was only employed for recording the IMU signals to create the dataset. To compute the model predictions, we leave aside the controller board Beagle Bone Black (BBB), which is more powerful for computation than Adafruit Feather M0. The gait phase detection, locomotion recognition algorithm, and prosthetic control program were embedded in this board. Additionally, a circuit that measures the battery voltage was designed to detect when the battery is in low energy. Besides, the wifi of the BBBB unit could be used for transmitting data to personal devices. This is appropriate for designing mobile applications so users can easily monitor or even control their prostheses.

### 3.2. Experimental protocol

**Figure 7:**
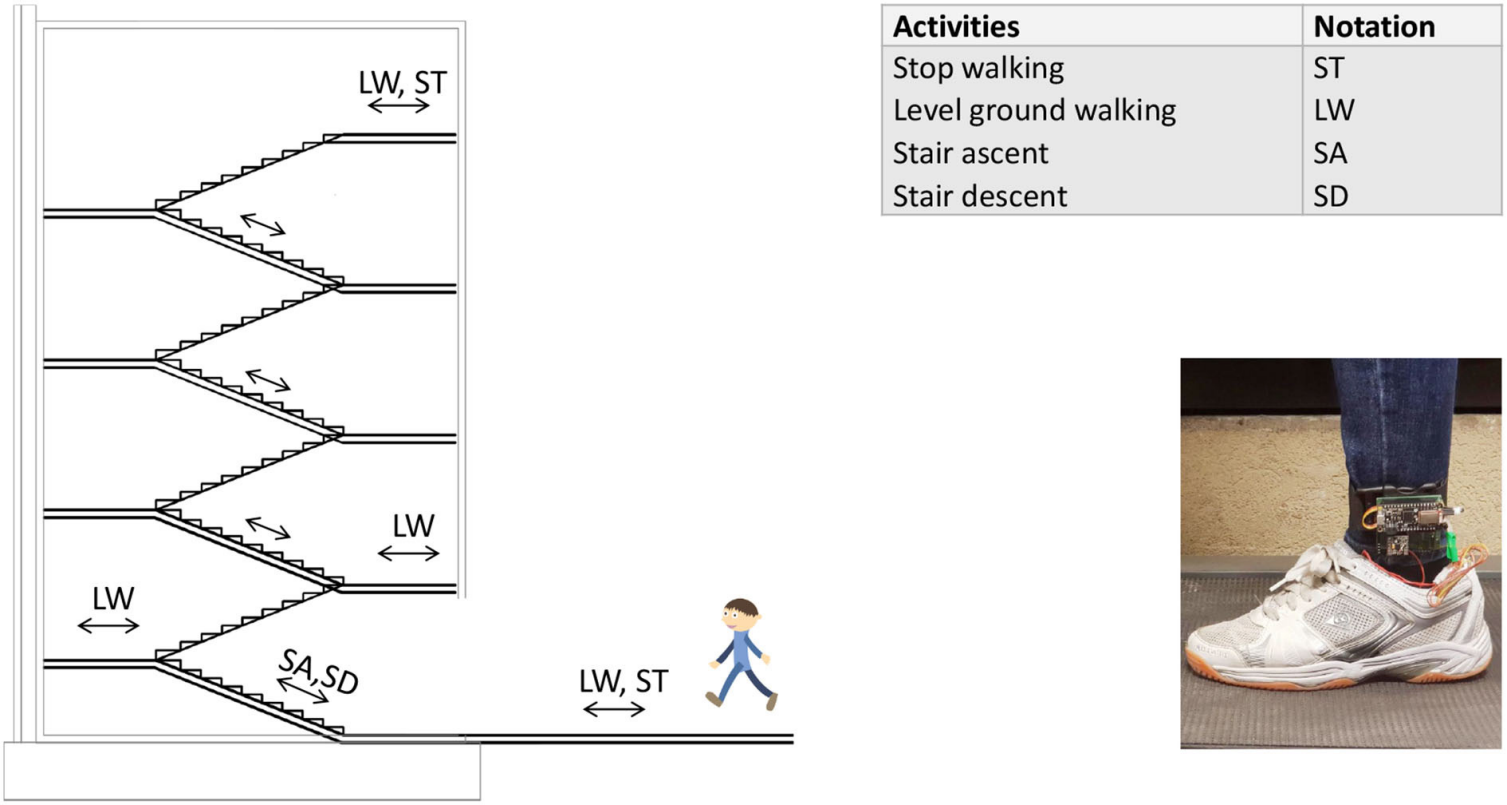
The experimental platform is set up with four locomotion modes and six mode transitions. Subjects wore the shoes equipped FSRs under the toe and the heel and performed tasks: Standing and walking on the flat ground for 5 min before walking up the stairs; climbing the stairs to the fourth floor; and walking downstairs to the starting position.

The experiment protocol is shown in Figure 7 and Table 1. The subjects who participated in this experiment were able-bodies since this obtains a larger dataset while helping to minimize the risks to amputees. Four healthy subjects aged 25 to 40 performed walking tasks with one IMU placed on the lower shank and two FSRs mounted under the sole of the participant. They were invited to perform different walking speeds in different scenarios. In the first scenario, subjects walked upstairs and downstairs of the four-floor building (5 repetitions). In the second scenario, they performed normal walking on flat ground, stopped, and repeated these activities in 5 min. For stair walking activity, they were asked to start walking on the ground, then climb the stairs to the fourth floor, and continue walking downstairs to the ground. In this scenario, when subjects transitioned between floors, they performed several steps of level walking. Subjects were required to stand 5 s at the start and the end of each trial. As a result, the data trials contained four locomotion modes (stop, level ground, upstairs, and downstairs) with four transitions between locomotion modes. We obtained one dataset of 229,074 samples belonging to 1,359 walking cycles on overage of 271.8 walking cycles per participant as shown in Table 2. We merged all IMU data signals recorded from participants to assemble a big dataset for training the network model. Integrating the dataset allowed the model to increase the chances of extracting the relevant features in different walking modes.

**Table 2:** The number of samples and cycles of the dataset.

**Subjects**	**Number of samples**	**Number of cycles**
Subject 1	44,432	398
Subject 2	69,508	345
Subject 3	54,099	449
Subject 4	61035	387
Dataset (all samples and cycles)	229,074	1,359

### 3.3. Off-line data analysis

FSRs were used to extract gait cycles by detecting the heel contact and the toe off the ground. The positions of FSRs are under the heel and the toe, and the location of the IMU is at the subject's lower shank, respectively. After gathering and organizing data, labeling was the last step for structuring massive amounts of data properly for machine learning. In our models, labeling was done automatically using simple thresholds and manually creating a structured dataset to train and deploy models. Compared to the other studies (Kotiadis et al., 2010; Boutaayamou et al., 2015; Wang et al., 2015; Maqbool et al., 2017), our models do not require complex pre-processing steps as they can deal with data with a high signal-to-noise ratio.

## 4. Results

### 4.1. Results and discussions

**Figure 8:**
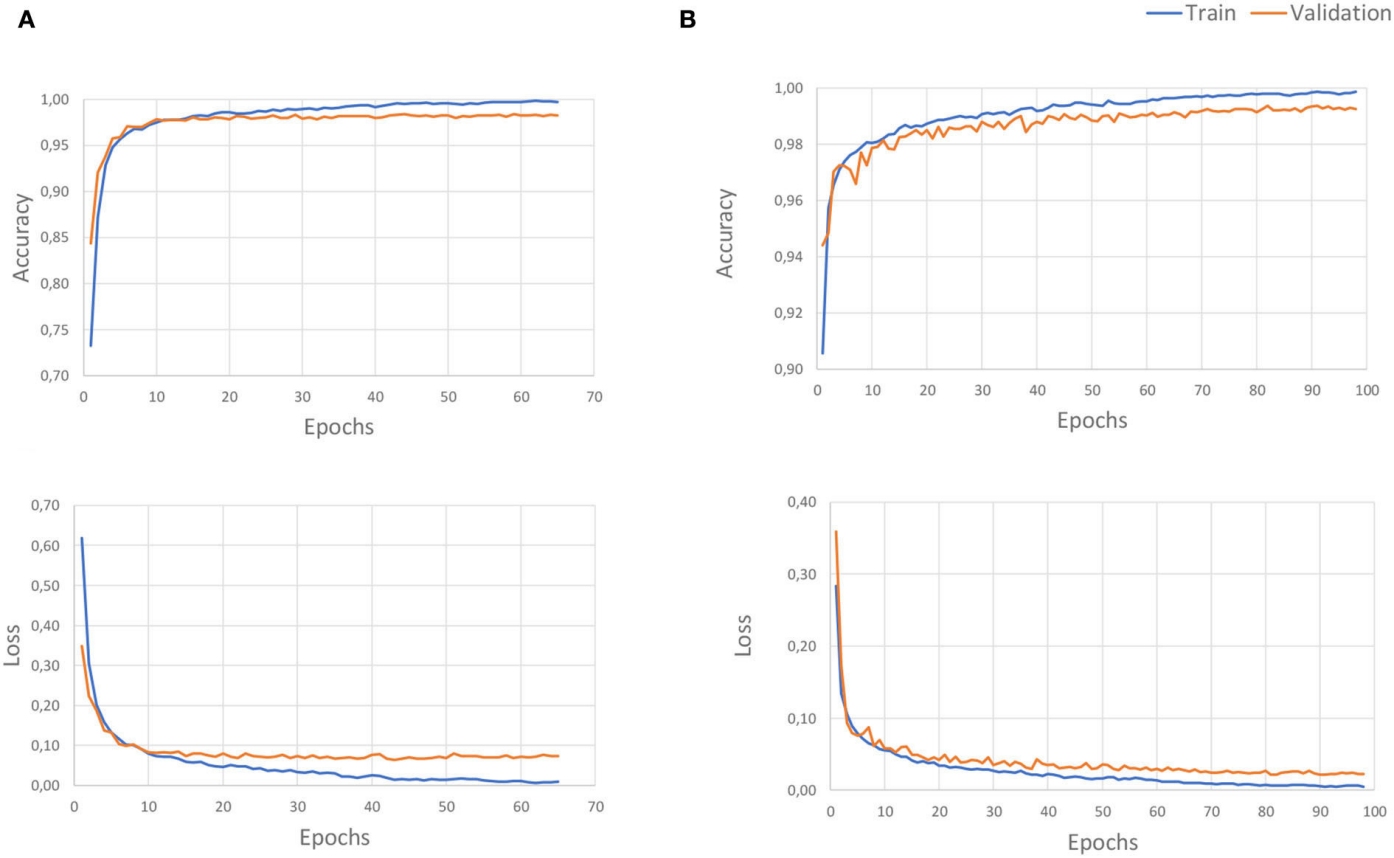
The overage accuracy and the loss of recognition learning curves: The **(A)** for the LSTM model, and **(B)** for the CNN model. The values were scaled in the range between 0 and 1, with the accuracy of 1 indicating a perfect prediction, the loss gradually decreases over time, and it tends close to zero to express perfect learning from the features of the dataset.

This study described four advanced deep learning models for locomotion detection. The models were learnt from the signals in three axes of angular rate and the acceleration of the foot. All signals were taken from healthy subjects walking at different speeds in four states walking on flat ground, up/downstairs, and stopped walking. The performances of four proposed methods are presented in this section using the evaluation described in Section 2.4 with separate 80% and 20% of all the datasets for training and validation, respectively. We used the accuracy and loss as two main parameters of the valuation metric. Figure 8 depicts the accuracy and the loss curves of LSTM and CNN models. The training loss and validation loss define the mean absolute error between the ground truth and predicted values for each epoch of these procedures (Mundt et al., 2021). The loss will be decreased over time and tend close to zero to express a perfect study from the features of the dataset. Besides, the validation loss parameter indicated over-fitting. The training will generally stop before reaching the specified maximum number of epochs to avoid over-fitting the data. After this point, the loss will not decrease further, and an optimum number of training epochs remains. Figure 8 displays that the LSTM model fits well after 80 epochs, faster than the CNN model which obtained stability for training after 140 epochs. The observation shows no over-fitting during the learning process for any model.

**Figure 9:**
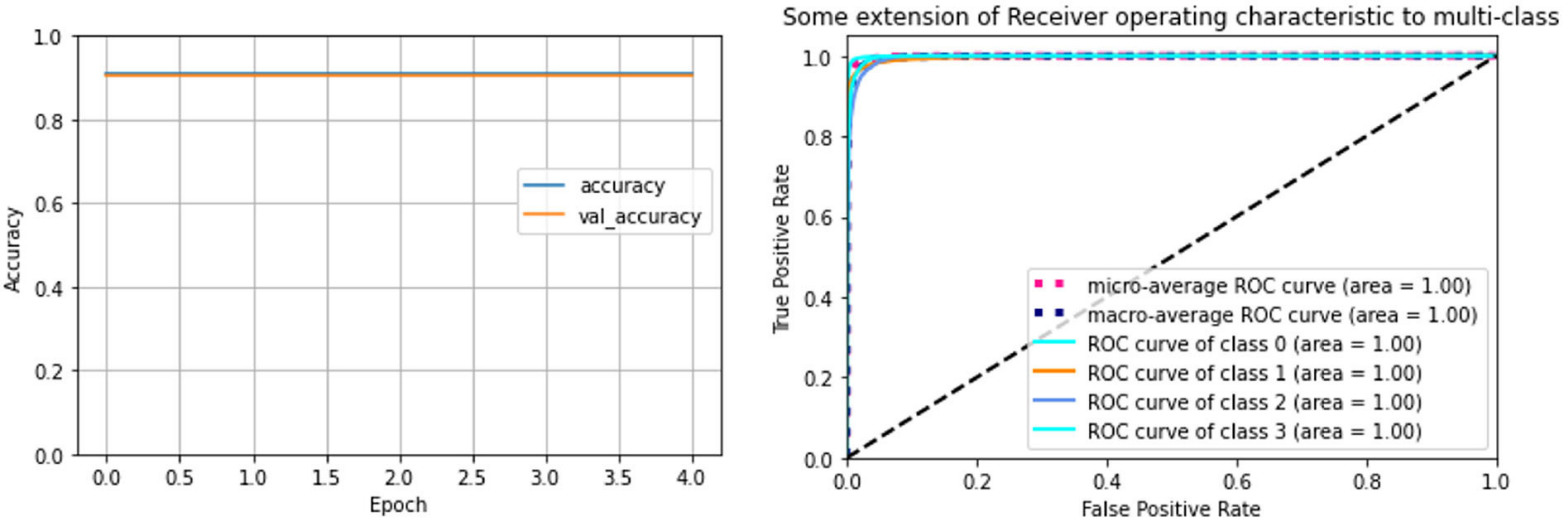
Performance of RFC model based on the accuracy and true and false positive rate. The values were scaled in the range between 0 and 1, with an accuracy of 1 indicating a perfect prediction. The receiver operating characteristic (ROC) curve tends close to 1 over time to express a perfect learning model.

**Figure 10:**
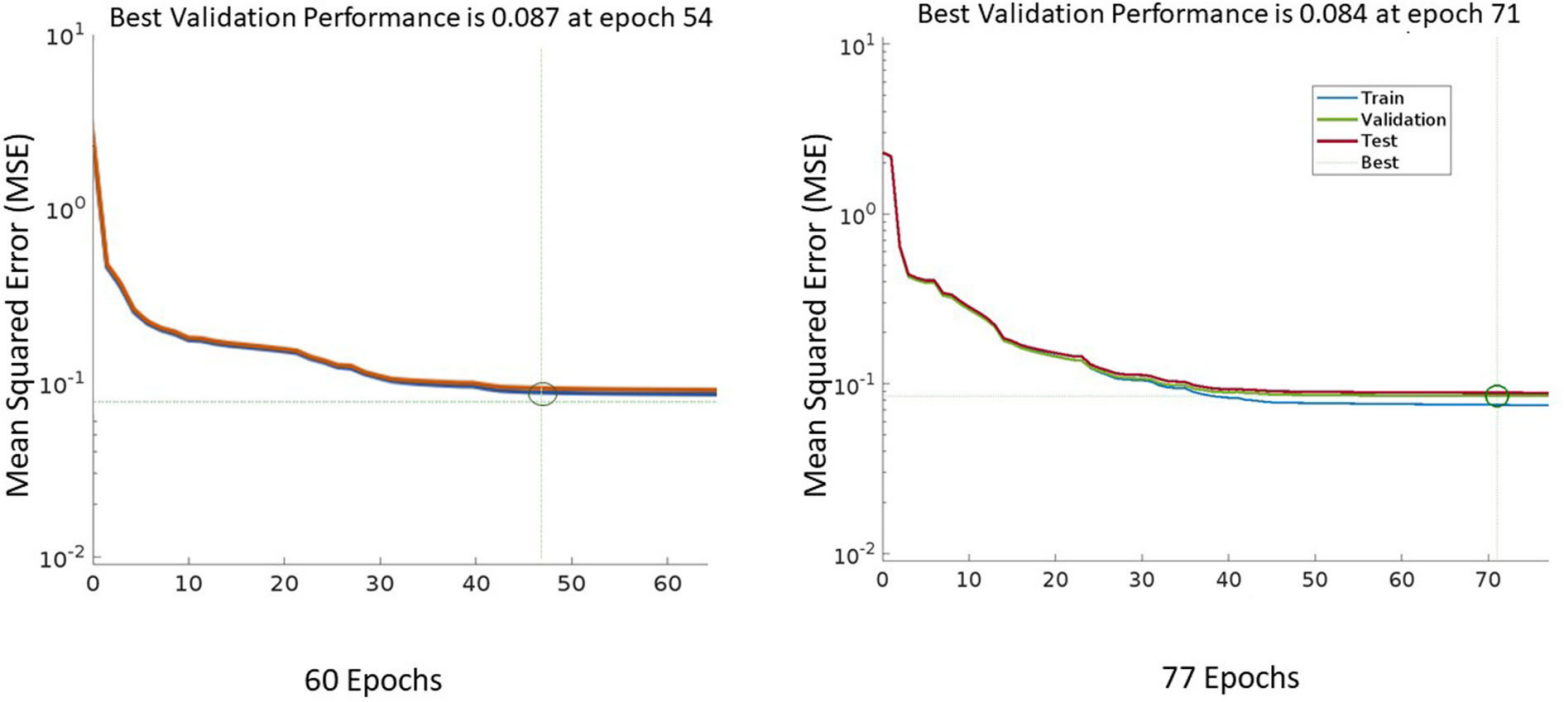
The performance Mean Squared Error (MSE) of the model learning with the window size of 100 time steps (on the right side) is higher than the performance of the model on the left side with the window size of 50 time steps. MSE is the loss function which is indicated by the loss value between the prediction and the ground truth.

Table 3 indicates the result performance obtained from four models over several runs of different window sizes of 50, 100, and 500 samples, with 10 ms for a sampling interval. Every sample is delayed by *n* times equal to the window size, and the window will be shifted one sampling step into the future. Overall, the CNN model obtained the highest accuracy of 99.60%, equivalently the smallest loss value reached 1.11%. By following the LSTM model, this model achieves over 98.68% of accuracy and an average loss value of 4.74%. These models outperformed other models such as the RFC model, achieving an average accuracy of 96%, and the RNN model, of 93.65% (Figures 9, 10). Though the RFC model is a simple classifier, the model performs very well, with the ROC being close to 1. The ROC curve is a plot of the true positive rate (sensitivity) vs. the false positive rate (1-specificity) as the threshold is varied. Figure 10 shows a perfect curve in the upper-left corner, with 100% sensitivity and 100% specificity. The data in Table 3 indicates that the window size impacts the accuracy of the DL model. The window size increases from 50 to 100 and 200, and the performance is also improved as the average accuracy increases slightly from 98.68 to 99.62%, and 99.83%, and the loss decreases from 4.75 to 1.18%, and 0.68% in the LSTM model; however, the computation of the model is expensive, since generated parameters as weight and bias matrices are bigger. This leads to a time-consuming output computation. We highlighted that the deep neural network CNN and LSTM with a window length of 50 samples are best suited for long-term prosthetic applications such as daily walking.

**Table 3:** The overage accuracy, loss, and computation delay of the long short-term (LSTM) and the computational neural network (CNN) models with different window sizes.

**Window length (Samples)**	**Accuracy (LSTM)**	**Accuracy (CNN)**	**Loss (LSTM)**	**Loss (CNN)**	**Delay (LSTM)**	**Delay (CNN)**	**Accuracy (RFC)**	**Accuracy (RNN)**	**Accuracy (RFC)**
50	98.68	99.60	4.75	1.12	133	110	96.08	93.65	≈92
100	99.62	99.99	1.82	0.02	376	344	–	95.29	–
200	99.83	99.99	0.68	0.01	768	273	–	–	–

### 4.2. Reference system and performance

Tables 4, 5 summarize the performance of different deep learning-based models and classification-based models for recognition and detection of locomotion mode transitions. The observation showed that existing studies employed types of different sensor, sensor placements, and methods for their systems and calculated the accuracy and the loss value to evaluate the performance of detection algorithms. To the best of our knowledge, it is difficult to compare our study with other studies as there are different setting standards for different systems. However, it was demonstrated that our methods performed effectively in terms of accuracy and the number of sensors. This study focused on selecting the optimal model structures, including efficient deep learning layers and efficient activation functions that helped the network to learn complex features in the data to obtain the outputs. Although our methods were implemented offline, our system provides an opportunity for real-time applications if we can reduce computation delays of hardware implementation on the device.

**Table 4:** Existing locomotion recognition based on deep learning methods applied on prostheses using inertial measurement unit (IMU) sensors.

**References**	**Sensors**	**Methods**	**Targeted**	**Accuracy**	**Problems**	**Online/Offline**
Feng et al. (2019)	Angle Sensor, Load cell	CNN	LW, SA, SD, RA, RD	92.1%	Recognition	Off
Wang et al. (2018)	Joint angles	LSTM	LW, SA, SD, ST, SIT	95%	Prediction	Off and on
Su et al. (2019)	3 IMUs	CNN	LW, SA, SD, RA, RD	89.2%	Recognition	Off
Lu et al. (2020b)	5 IMUs	CNN	LW, SA, SD, RA, RD	95%	Recognition	Off
Hu et al. (2018b)	5 IMUs	CNN	LW, SA, SD, RA, RD	95%	Recognition	Off
Woodward et al. (2016)	1 x IMU 6 x 1 Load cell 2 x Joint angle 2 x Joint Current 2 x Joint velocity	ANN	LW, SA, SD, RA, RD	98.9%	Prediction	Off
Narayan et al. (2021)	7 x IMU	CNN	Sit, Stand, Walk, Unknown, Straight, Turn, Curved (left, right) Stair (up, down)	94.34%	Prediction	On
Our methods	1 IMU	CNN, LSTM, RFC, RNN	LW, SA, SD, ST	99.60% 98.68% 96.98% 95.29%	Recognition Recognition Prediction Prediction	Off

**Table 5:** Existing locomotion recognition based on machine learning methods using signals from many sensor types.

**References**	**Sensors**	**Methods**	**Targeted**	**Accuracy**	**Problems**	**Online/Offline**
Han et al. (2021)	1 x IMU	Decision Tree Structure IBPNN	LW, SA, SD, RA, RD, ST, SIT	96.71%	Recognition	Off
Gong et al. (2020)	3 IMUs	BPNN	LW, SA, SD, RA, RD, ST	98.4%	Recognition	Off and On
Billah et al. (2019)	3 x IMU 1 x FSR	Decision Tree	LW, SA, SD	≈98%	Prediction	Off and On
Liu et al. (2015)	1 x IMU 1 x Laser with Camera 6 DOF load cell 1 x EMG electrode	Decision Tree	LW, RA, SA, RD, SD	≈99%	Prediction	Off
Bhakta et al. (2020)	2 x encoders 3 x IMUs 1 x Loadcell	XGBoost	LW, RA, RD, SA, SD	89.89//96.19%	Recognition	Off
Stolyarov et al. (2017)	IMU	LDA	LW, SA, SD, RA, RD	94.1%	Prediction	Off
Chen et al. (2015)	4 x Pressure	LDA	LW, SA, SD, OBS, SIT	98.4%	Recognition	Off
Du et al. (2012)	9 x EMG 1 x 6 axis Pressure	LDA	LW, SA, SD, RA, RD	98%	Prediction	Off
Liu et al. (2016)	8 x EMG 1 x Loadcell 1 x IMU 1 x Laser	LDA	LW, SA, SD, RA, RD	98%	Prediction	Off and on
Tkach and Hargrove (2013)	4 x EMG 1 x Joint angle 1 x IMU 1 x Joint velocity 1 x Joint current	LDA	LW, SA, SD, RA, RD	96%	Prediction	Off
Ai et al. (2017)	4 x EMG 1 x IMU	VSM	LW, SA, SD, ST, SQ	≥95%	Recognition	Off
Huang et al. (2011)	11 x EMG 6 x Loadcell Pressure	SVM	LW, SA, SD, RA, RD, OBS	100%	Prediction	Off
Huang et al. (2011)	4 x IMU 3 x 2 Pressure	SVM	LW, SA, SD, RA, RD	98.4%	Prediction	Off and on
Zhang et al. (2011)	1 x IMU 6 x Load cell 1 x EMG	SVM	LW, SA, SD, RA, RD, ST, SIT	95%	Prediction	Off and on
Zheng and Wang (2016)	2 x IMU 1 x Load cell 4 x Pressure 1 x Joint angle 6 x Capacitive	SVM	LW, SA, SD, RA, RD, ST	95%	Prediction	Off
Mai et al. (2018)	2 IMUs	SVM	LW, SA, SD, RA, RD	99.1%	Recognition	Off and On
Zhou et al. (2019)	2 x IMU 2 x Load cell 1 x Joint angle	SVM	LW, SA, SD, RA, RD, ST	95%	Prediction	Off and on

## 5. Conclusion

Active leg prostheses often switch control strategies for different locomotion modes. Therefore, we introduced a locomotion mode recognition system suited for a lower limb prosthesis with only one IMU. This system is durable, low-cost, and easily implemented to avoid direct contact with the human body, and utilizes a minimal source of information. The proposed methodologies recognize four locomotion modes: LW, ST, SA, and SD. The transitions between these modes are easy to compute from the difference between the modes for prosthetic applications. Our previous study proposed a gait phase prediction method with the same system. We aimed to fully control upcoming active prostheses by combining phase gait prediction, locomotion mode recognition into the prosthetic control. The real-time commutation of deep learning models often costs time, with a significant delay generated from the calculation of vast dimensions of the weight and neurons if the deep learning models are created with many layers and neurons. Though our experiments were purely offline, the computational cost was not a concern. We can expect that computations of proposed models do not cost much time because we first learn the models on a personal computer and then make the predictions on the controller board. It is important to note that performing a deep learning model requires a specialized hardware. Hence, we used the board Adafruit Feather M0 (ARM M0 Cortex, 48 Mhz), presented in Section 3.1, for recording the data only. Furthermore, when embedding heavy mathematical computations, we planned to use the board Beagle Bone Black (BBB), which is known to outperform Adafruit Feather M0. The second reason is that the models built in our system are not computationally heavy as we used only a 6-dimensional vector of signal inputs from one IMU. In the future work, we aim to overcome these limitations. We strive toward a real-time performance that is similar to the offline performance by reducing the cost of model computation to take full control of the prosthesis while it is being worn.

## Data availability statement

The raw data supporting the conclusions of this article will be made available by the authors, without undue reservation.

## Ethics statement

The studies involving human participants were reviewed and approved by Ethics Committee Universitair Ziekenhuis Brussel-Vrije Universiteit Brussel. The patients/participants provided their written informed consent to participate in this study.

## Author contributions

HV, JG, H-LC, TV, DD, and BV conceptualized the work. HV and DD designed the methods and conducted the experiments. HV analyzed the data and wrote the original manuscript. H-LC, TV, DD, BV, and JG modified and supervised the writing of the manuscript. All authors contributed to the article and approved the submitted version.

## Funding

This project was partly supported by the Innoviris' Talaris project, the AI Flanders program, and Vietnamese Government for university and college lecturers on doctoral training during 2010–2020.

## Conflict of interest

The authors declare that the research was conducted in the absence of any commercial or financial relationships that could be construed as a potential conflict of interest.

## Publisher's note

All claims expressed in this article are solely those of the authors and do not necessarily represent those of their affiliated organizations, or those of the publisher, the editors and the reviewers. Any product that may be evaluated in this article, or claim that may be made by its manufacturer, is not guaranteed or endorsed by the publisher.
